# p53 coordinates DNA repair with nucleotide synthesis by suppressing PFKFB3 expression and promoting the pentose phosphate pathway

**DOI:** 10.1038/srep38067

**Published:** 2016-11-30

**Authors:** Derek A. Franklin, Yizhou He, Patrick L. Leslie, Andrey P. Tikunov, Nick Fenger, Jeffrey M. Macdonald, Yanping Zhang

**Affiliations:** 1 Department of Radiation Oncology and Lineberger Comprehensive Cancer Center, University of North Carolina at Chapel Hill, Chapel Hill, North Carolina, USA; 2 Department of Pharmacology, School of Medicine, University of North Carolina at Chapel Hill, Chapel Hill, NC 27514, USA; 3 Curriculum in Genetics and Molecular Biology, School of Medicine, University of North Carolina at Chapel Hill, Chapel Hill, NC 27514, USA; 4 UNC Metabolomics Laboratory, School of Medicine, University of North Carolina at Chapel Hill, Chapel Hill, NC 27514, USA; 5 Jiangsu Center for the Collaboration and Innovation of Cancer Biotherapy, Cancer Institute, Xuzhou Medical College, Xuzhou, Jiangsu 221002, China

## Abstract

Activation of p53 in response to DNA damage is essential for tumor suppression. Although previous studies have emphasized the importance of p53-dependent cell cycle arrest and apoptosis for tumor suppression, recent studies have suggested that other areas of p53 regulation, such as metabolism and DNA damage repair (DDR), are also essential for p53-dependent tumor suppression. However, the intrinsic connections between p53-mediated DDR and metabolic regulation remain incompletely understood. Here, we present data suggesting that p53 promotes nucleotide biosynthesis in response to DNA damage by repressing the expression of the phosphofructokinase-2 (PFK2) isoform 6-phosphofructo-2-kinase/fructose-2,6-biphosphatase 3 (PFKFB3), a rate-limiting enzyme that promotes glycolysis. PFKFB3 suppression increases the flux of glucose through the pentose phosphate pathway (PPP) to increase nucleotide production, which results in more efficient DNA damage repair and increased cell survival. Interestingly, although p53-mediated suppression of PFKFB3 could increase the two major PPP products, NADPH and nucleotides, only nucleotide production was essential to promote DDR. By identifying the novel p53 target PFKFB3, we report an important mechanistic connection between p53-regulated metabolism and DDR, both of which play crucial roles in tumor suppression.

The transcription factor p53 regulates the expression of genes involved in many cellular processes, including cell cycle arrest, senescence, apoptosis, DNA damage repair, and metabolism^1^,^2^,^3^. Despite its ability to regulate a seemingly diverse array of pathways, p53 activation regularly exerts a net tumor suppressive effect. p53 tumor suppression is demonstrated by the homozygous deletion of p53 in mice, which results in the rapid development of tumors^4^. Consistent with its importance in tumor development, p53 has been confirmed as the most commonly mutated gene across all forms of cancer^5^. p53 is colloquially referred to as the “guardian of the genome” for its role in inducing cell cycle arrest in the presence of DNA damage^6^. Specifically, p53-dependent cell cycle arrest in the G1 phase through the induction of *CDKN1A* (p21) expression prevents the incorporation of mutations into the genome^7^,^8^. p53-dependent G1 arrest is a necessary checkpoint that allows the cell an opportunity to either repair DNA damage before re-entering the cell cycle or initiate apoptosis when the damaged DNA is beyond repair.

One common hypothesis regarding the tumor suppressive function of p53 has been that the canonical effects of p53 activation (i.e., cell cycle arrest, senescence, and apoptosis) are more important and are thus of more interest from a clinical perspective. However, less well-studied p53-regulated pathways, such as metabolism and DNA damage repair (DDR), are gaining recognition as being equally necessary for p53-dependent tumor suppression. These non-canonical p53-regulated pathways are currently being evaluated for their relative importance in p53-dependent tumor suppression. Multiple *in vivo* studies over recent years have suggested that p53 exerts significant tumor suppressor activity in the absence of cell cycle arrest, senescence, and apoptosis; therefore, efforts to further characterize non-canonical functions of p53 are needed^9^,^10^.

Two branches of the p53 stress response that likely contribute to its tumor suppressive effects are genes involved in metabolism and DDR. As a metabolic regulator, p53 inhibits glycolysis at multiple points by repressing the expression of glucose transporters GLUT1 and GLUT4 as well as pyruvate dehydrogenase kinase 2 (PDK2)^11^,^12^,^13^. p53 also induces the expression of the glycolytic inhibitor TIGAR (tp53-induced glycolysis and apoptosis regulator)^14^. As a DDR regulator, p53 directly regulates the expression of the nucleotide excision repair genes *XPC* and *DDB2*, which contribute to the repair of DNA lesions that occur in response to UV irradiation^15^,^16^,^17^. Similarly, p53 contributes to the maintenance of the deoxyribonucleotide pool, which is an important component of DDR, as nucleotide shortage or imbalance can result in incorrect base insertion^18^. Indeed, in response to DNA damage, p53 up-regulates the expression of the ribonucleotide reductase p53R2 to facilitate accurate nucleotide incorporation through the conversion of ribonucleotides (rNTPs) to deoxyribonucleotides (dNTPs)^19^. Interestingly, despite the ability of p53 to increase dNTPs at the expense of rNTPs, p53 has also been reported to inhibit the pentose phosphate pathway (PPP), which is the biosynthetic pathway through which rNTP and dNTP precursors are produced^20^,^21^. Importantly, p53-mediated inhibition of the PPP is dependent on direct binding of p53 and glucose-6-phosphate dehydrogenase (G6PDH) in the cytoplasm rather than p53 transcriptional regulation^21^. Nonetheless, whether the p53-dependent regulation of metabolism could play a role in maintaining sufficient nucleotide levels through *de novo* biosynthesis in response to DNA damage remains unknown. In this study, we identify the PFK2 isoform 6-phosphofructo-2-kinase/fructose-2,6-biphosphatase 3 (PFKFB3), a potent stimulator of glycolysis, as a novel p53 suppression target and seek to determine the role of p53-PFKFB3 regulation in the context of p53 stress response.

## Results

### PFKFB3 is a p53 suppression target

To identify novel p53 target genes, we analyzed a previously described mRNA microarray database^22^, which is based on mouse embryonic fibroblast (MEF) cells expressing an inducible p53-estrogen receptor (p53ER; *p53*^*ER/−*^ MEF cells) fusion protein. The p53ER fusion protein is inactive in the absence of the tamoxifen derivative 4-hydroxytamoxifen (4-OHT), which allows for p53 activation independent of damage or other cellular stress. Using this system, we identified *Pfkfb3* as a novel p53 suppression target gene. PFKFB3 is a bifunctional enzyme that catalyzes the rate-limiting glycolysis step of generating fructose-(2,6)-bisphosphate (F2,6BP) through its N-terminal kinase domain, which is significantly more active than its C-terminal phosphatase domain. F2,6BP is a potent allosteric activator of PFK1; therefore, PFKFB3-mediated F2,6BP generation dramatically increases glycolytic flux^23^,^24^.

**Figure 1:**
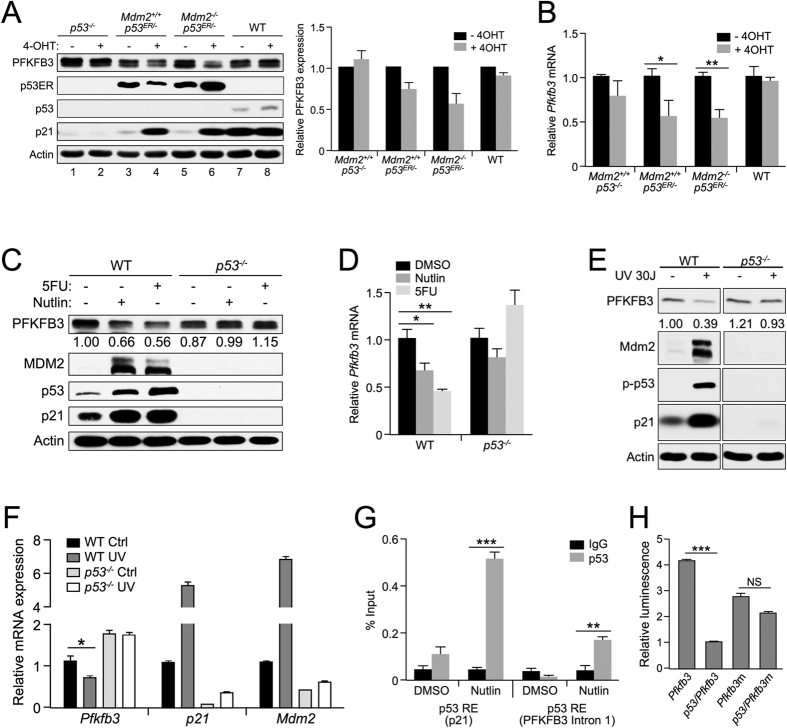
PFKFB3 is a p53 suppression target. (**A**) *p53*^−/−^, *Mdm2*^*+/+*^;*p53*^*ER/−*^, *Mdm2*^−/−^;*p53*^*ER/−*^, and WT MEF cells were treated (+/−) 100 nM 4-OHT for 24 h prior to lysis and immunoblotting. Densitometry analysis using Image J software was used to compare PFKFB3 expression after normalization to actin for 3 independent experiments. The PFKFB3 expression level observed in the (-)4OHT sample for each cell line was set to 1 for normalization. (**B**) Relative mRNA levels of *Pfkfb3* were determined by qRT-PCR in *p53*^−/−^, *Mdm2*^*+/+*^;*p53*^*ER/−*^, *Mdm2*^−/−^;*p53*^*ER/−*^and WT MEF cells 12 h after treatment with 100 nM 4-OHT. Levels were normalized to actin, and the levels of *Pfkfb3* detected in the vehicle-treated samples for each cell line was set to 1 for normalization. (WT-ER p = 0.029 ^−/−^ ER p = 0.003 n = 3 for each sample) (**C**) WT and *p53*^−/−^ MEF cells were treated (+/−) 10 μM nutlin or 10 μM 5-FU for 24 h prior to immunoblotting for the indicated proteins. (**D**) Relative mRNA levels of *Pfkfb3* were determined by qRT-PCR in *p53*^−/−^ and WT MEF cells 12 h after treatment with DMSO, 10 μM nutlin or 10 μM 5-FU. Levels were normalized to actin, and the levels of *Pfkfb3* observed in the DMSO-treated samples for each cell line were set to 1. (WT-Nutlin p = 0.016;WT-5FU p = 0.001 n = 3 for each sample) (**E**) WT and *p53*^−/−^ MEF cells were treated with 30 J/m^2^ UV for 24  h followed by immunoblotting for protein expression. (**F**) Relative mRNA levels of *Pfkfb3, p21*, and *Mdm2* were determined by qRT-PCR in WT and *p53*^−/−^ MEF cells 24  h after treatment with 40 J/m^2^ UV. Levels were normalized to actin, and the relative levels of each mRNA detected in the untreated cells was set to 1. (p = 0.0376 n = 6) **G**) Chromatin immunoprecipitation of the putative *Pfkfb3* p53RE located within intron 1 and the *p21* p53RE in U2OS cells 12 h after 10 μM nutlin treatment. (PFKFB3 p = 0.002; p21 p < 0.001 n = 3) (**H**) Exogenous overexpression of *Pfkfb3* Intron 1 p53RE-luciferase (*Pfkfb3*) and mutant *Pfkfb3* Intron 1 p53RE-luciferase (*Pfkfb3m*) in H1299 cells. Relative luminescence is the increase in luminescence signal compared with the vector control. (p = 0.0002 n = 3).

To confirm the effect of p53 on PFKFB3 expression, we analyzed PFKFB3 protein expression levels in *Mdm2*^*+/+*^;*p53*^*ER/−*^ and *Mdm2*^−/−^;*p53*^*ER/−*^ MEF cells in the presence or absence of 4-OHT. Consistent with the microarray results, *Mdm2*^*+/+*^;*p53*^*ER/−*^ and *Mdm2*^−/−^;*p53*^*ER/−*^ MEF cells exhibited decreased PFKFB3 protein expression in the presence of 4-OHT (Fig. 1A, lanes 4 and 6). Importantly, WT and *p53*^−/−^ MEF cells exhibited no response to 4-OHT (Fig. 1A, lane 2 and 8). Moreover, quantitative RT-PCR (qRT-PCR) analysis showed approximately 50% decrease in *Pfkfb3* mRNA levels after 4-OHT treatment (Fig. 1B). To verify that PFKFB3 down-regulation is directly related to p53 activation, we treated *p53*^*ER/−*^ MEF cells with 4-OHT and increasing amounts of nutlin, a small molecule activator of p53. Consistently, we observed a dose-dependent decrease in PFKFB3 expression only when p53 was activated with 4-OHT (Figure S1).

To determine whether PFKFB3 suppression is observed in a non-p53ER system, we treated WT and *p53*^−/−^ MEF cells with nutlin and the nucleoside analog 5-fluorouracil (5-FU), two activators of p53, and found that nutlin and 5-FU induce p53-dependent PFKFB3 suppression (Fig. 1C). This decrease in protein expression is likely dependent upon p53 transcriptional regulation, as *Pfkfb3* mRNA levels were significantly decreased after nutlin and 5-FU treatment in WT but not *p53*^−/−^ MEF cells (Fig. 1D). To determine whether p53 regulates PFKFB3 in response to other forms DNA damage, we analyzed PFKFB3 expression in WT and *p53*^−/−^ MEF cells after exposure to 30 J/m^2^ of UV irradiation. In agreement with our results after 5-FU-induced DNA damage, PFKFB3 protein expression decreased in WT but not *p53*^−/−^ MEF cells in response to UV irradiation (Fig. 1E). Furthermore, the decrease in PFKFB3 expression inversely correlated with increased expression of MDM2 and p21, two archetypal p53 transactivation targets, and increased levels of phosphorylated p53. Similarly, we observed a UV-induced decrease in *Pfkfb3* mRNA levels and increases in *p21* and *Mdm2* mRNA levels, suggesting that p53 transcriptionally regulates *Pfkfb3* in response to both UV- and 5-FU-induced DNA damage (Fig. 1F).

To determine whether p53 directly suppresses *Pfkfb3*, we searched the *Pfkfb3* promoter region and identified a potential p53 response element (p53RE) consistent with the consensus p53 binding sequence^25^ (Figure S2). In support of a direct suppression mechanism, chromatin immunoprecipitation assays probing for p53 binding within *PFKFB3* showed specific binding of p53 to a putative *PFKFB3* p53RE in intron 1 (Fig. 1G). To test the functionality of this putative p53RE, we constructed a luciferase reporter under the control of the putative *PFKFB3* p53RE. When this reporter was co-expressed with p53 in p53-null H1299 cells, we observed a significant (p = 0.0002) decrease in luciferase signal (Fig. 1H). Moreover, when the essential central C and G nucleotides from the p53RE consensus sequence were mutated, the p53-dependent repression of luciferase activity was abolished (Fig. 1H).

### PFKFB3 down-regulation inhibits glycolysis

**Figure 2:**
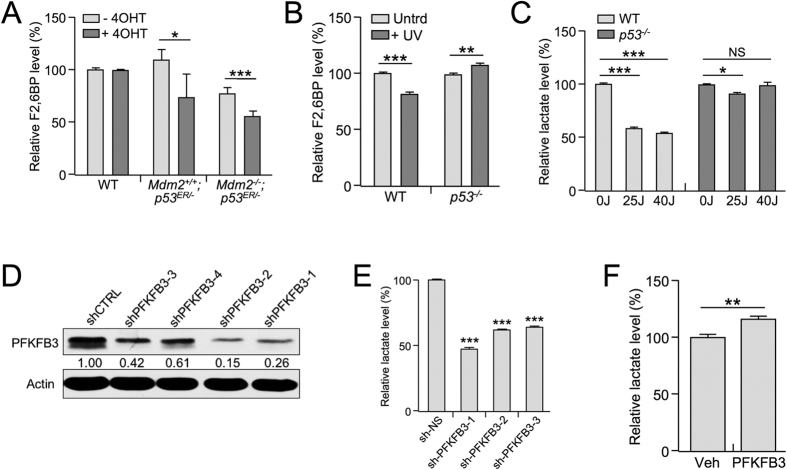
PFKFB3 down-regulation inhibits glycolysis. (**A**) Fructose-(2,6)-bisphosphate (F2,6BP) levels were determined in WT, *Mdm2*^*+/+*^;*p53*^*ER/−*^ and *Mdm2*^−/−^;*p53*^*ER/−*^ MEF cells in the presence or absence of 4-OHT after 24 h. The amount of F2,6BP detected in the vehicle-treated WT MEF cells was set at 100%. (WT ER p = 0.0218; ^−/−^ ER p = 0.0006 n = 5) (**B**) Fructose-(2,6)-bisphosphate (F2,6BP) levels were assayed in WT and *p53*^−/−^ MEF cells 24 h after treatment with 40 J/m^2^ UV, and the amount of F2,6BP detected in the untreated samples for each cell line was set to 100%. (WT p < 0.0001; ^−/−^ p = 0.0013 n = 5) (**C**) Lactate levels were measured in WT and *p53*^−/−^ MEF cells 24  h after treatment with 0 J/m^2^, 25 J/m^2^, or 40 J/m^2^ UV, and the amount of lactate detected in the untreated samples for each cell line was set to 100%. (WT 0/25J p < 0.0001; WT 0/40J p < 0.0001; ^−/−^ 0/25J p = 0.0308 n = 3) (**D**) Expression levels of PFKFB3 were determined by western blot for *p53*^−/−^ MEF cells stably infected with 4 different shRNA constructs targeting *Pfkfb3*. (**E**) Extracellular lactate levels were measured in *p53*^−/−^ MEF cells transduced with 3 unique shRNA constructs specifically targeting *Pfkfb3* (sh-PFKFB3) and compared with cells transduced with the non-specific scrambled control. The non-specific scrambled control samples were designated as 100% (#1 p < 0.0001; #2 p < 0.0001, #3 p < 0.0001 n = 3). (**F**) Lactate levels were measured 24 h after exogenous overexpression of PFKFB3 in U2OS cells, and the lactate level detected in the control cells was set to 100%. (p = 0.022 n = 3).

To determine whether the p53-dependent inhibition of PFKFB3 expression affects the level of the PFKFB3 product F2,6BP, a potent glycolysis-promoting molecule, we analyzed the concentration of F2,6BP upon p53 activation. We found a decrease in the concentration of F2,6BP in response to p53 activation in *p53*^*ER/−*^ MEF cells (Fig. 2A), which is consistent with reduced PFKFB3 expression (Fig. 1A,B). Similarly, WT MEF cells exhibited decreased levels of F2,6BP in response to UV irradiation, whereas *p53*^−/−^ MEF cells showed a slight p53-independent increase in F2,6BP levels (Fig. 2B). The mechanism behind this p53-independent increase in F2,6BP is unknown, but PFKFB3 is regulated by several kinases that are likely to respond to UV-induced cell stress, including p38, AMPK, and PKC, which in the absence of p53 may play a more prominent role in F2,6BP regulation^26^,^27^,^28^. Additionally, MEF cells exhibited a p53-dependent decrease in lactate, which is indicative of decreased glycolytic production, in response to UV irradiation (Fig. 2C). Previous studies analyzing the effects of RNAi-mediated PFKFB3 suppression reported decreased glycolytic flux, as indicated by decreased production of lactate^29^,^30^. Correspondingly, in response to shRNA-mediated down-regulation of PFKFB3, lactate production decreased (Fig. 2D,E). Conversely, exogenous expression of PFKFB3 resulted in increased lactate production (Fig. 2F). Collectively, our results suggest that p53-mediated suppression of *Pfkfb3* contributes to the suppression of glycolysis.

### PFKFB3 down-regulation facilitates DNA damage repair and survival

**Figure 3:**
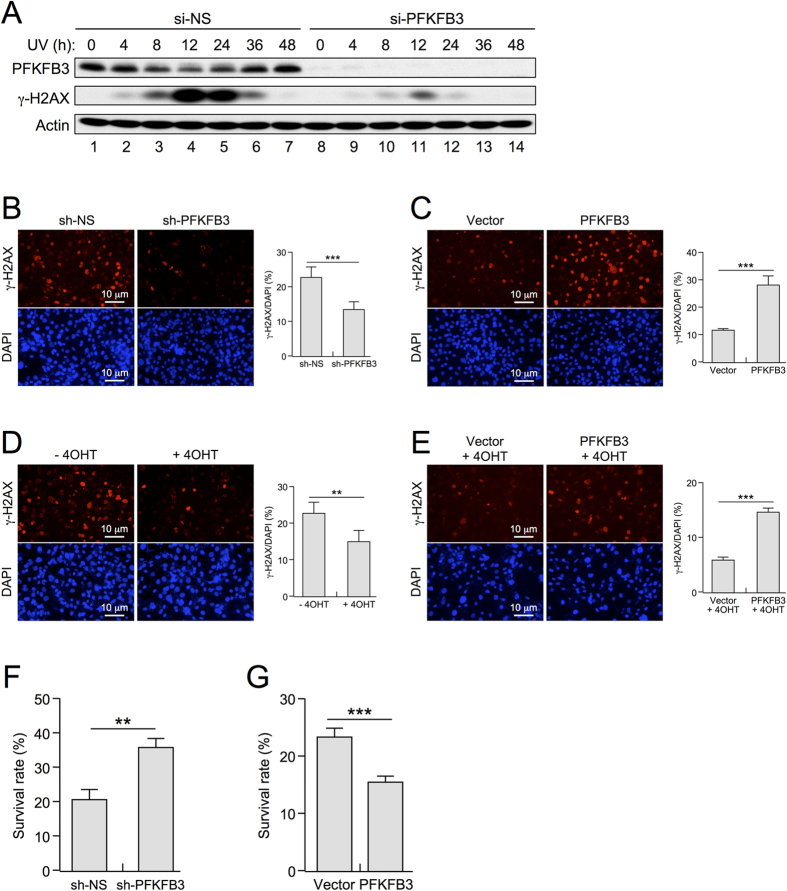
PFKFB3 down-regulation facilitates DNA damage repair and survival. (**A**) si-NS and si-PFKFB3 pretreated U2OS cells were treated with 15 J/m^2^ UV for varying amounts of time prior to immunoblotting for protein expression. (**B**) U2OS cells infected with lentiviral particles expressing shRNA constructs specific for PFKFB3 (sh-PFKFB3) and non-specific scrambled control (sh-NS) were treated with caspase inhibitor QVD-OPh for 30 minutes prior to treatment with 25 J/m^2^ UV to prevent apoptosis. Fresh medium containing QVD-OPh was added after treatment, and the cells were incubated at 37 °C for 48 hours prior to fixation and staining for the DNA damage marker γ-H2AX along with DAPI to visualize the total number of nuclei present. (p < 0.0001, n = 6) (**C**) *Mdm2*^*+/+*^;*p53*^*ER/−*^ MEF cells stably infected with lentiviral particles harboring PFKFB3-GFP or GFP constructs were treated with caspase inhibitor QVD-OPh for 30 minutes prior to treatment with UV 10 J/m^2^ to prevent apoptosis. Fresh medium containing QVD-OPh was added after treatment, and the cells were incubated at 37 °C for 48 hours prior to fixation and staining for the DNA damage marker γ-H2AX along with DAPI to visualize the total number of nuclei present. (p < 0.0001 n = 5) (**D**) *Mdm2*^*+/+*^;*p53*^*ER/−*^ MEF cells treated (+/−) 4-OHT were processed and analyzed as in panel C. (p = 0.0041  n = 7) (**E**) *Mdm2*^*+/+*^;*p53*^*ER/−*^ MEF cells stably infected with lentiviral particles harboring PFKFB3-GFP or GFP constructs were treated with 4-OHT before processing and analyzing as in panel C. (p < 0.0001  n = 5) (**F**) U2OS cells infected with lentiviral particles expressing shRNA constructs specific for PFKFB3 (sh-PFKFB3) and non-specific scrambled control (sh-NS) were irradiated with UV 40  J/m^2^. Twenty-four hours after treatment, the cells were trypsinized and counted using a Bio-Rad TC20 automated cell counter (p = 0.0036  n = 3) (**G**) U2OS cells overexpressing PFKFB3 by adenoviral infection were processed and analyzed as in panel F (p = 0.0033  n = 3).

The suppression of PFKFB3 and inhibition of lactate production in response to UV-induced DNA damage suggest that the suppression of glycolysis could play a role in DNA damage repair. To test this hypothesis, we analyzed the levels of the DNA damage marker γ-H2AX in U2OS cells subjected to UV irradiation. Consistent with a potential role in promoting DDR, the levels of PFKFB3 showed an inverse correlation with γ-H2AX signal. After UV irradiation, PFKFB3 levels begun to decrease between 4 and 8 h, reached the lowest observed levels at 12 h, and recovered to basal levels by 48 h (Fig. 3A, lanes 1–7). More importantly, upon knockdown of *PFKFB3* by siRNA, γ-H2AX levels were sharply reduced at all time points (Fig. 3A, lanes 8–14), indicating that down-regulation of PFKFB3 affects DNA damage accumulation. Immunofluorescence imaging of U2OS cells treated with UV irradiation also showed a direct correlation between PFKFB3 expression and γ-H2AX levels (Fig. 3B). Consistently, exogenous overexpression of PFKFB3 resulted in a nearly 3-fold increase in γ-H2AX-positive cells after UV irradiation in *p53*^*ER/−*^ MEF cells in the absence of 4-OHT (Fig. 3C).

Next, we analyzed inducible *p53*^*ER/−*^ MEF cells to determine the relative contributions of p53 activation in the context of DDR. As expected, 4-OHT-induced p53 activation resulted in significantly lower levels of γ-H2AX (p = 0.0041) after UV irradiation (Fig. 3D). Interestingly, exogenous overexpression of PFKFB3 was sufficient to abrogate the DNA repair function of p53 (Fig. 3E), suggesting that in the absence of PFKFB3 suppression (and hence glycolytic repression) following DNA damage, cells cannot effectively repair damaged DNA. Additionally, the decrease in γ-H2AX foci associated with cells in which *PFKFB3* expression was silenced correlated with a survival benefit in response to UV-induced DNA damage (Fig. 3F and Figure S3). Consistently, exogenous overexpression of PFKFB3 negatively affected cell survival in response to UV irradiation (Fig. 3G). Thus, p53-mediated down-regulation of *PFKFB3* expression after UV irradiation plays an essential role in DNA repair and cell survival.

### UV irradiation induces p53-dependent down-regulation of *PFKFB3* and up-regulation of nucleotide levels

**Figure 4:**
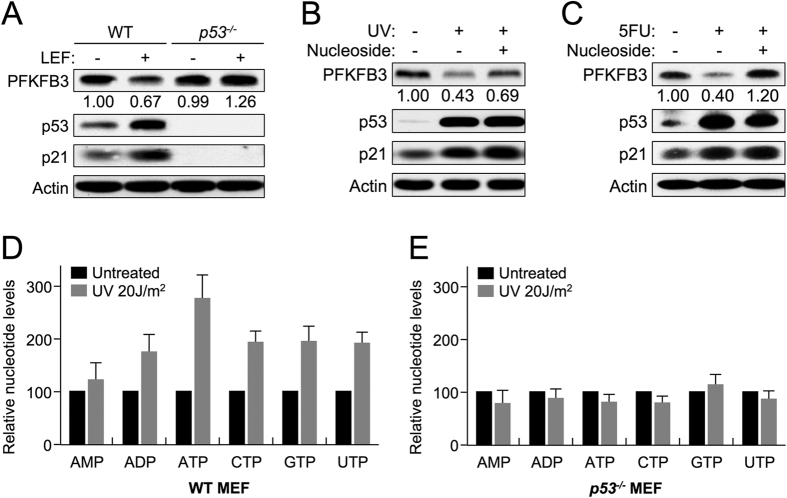
UV irradiation induces p53-dependent down-regulation of *PFKFB3* and up-regulation of nucleotide levels. (**A**) WT and *p53*^−/−^ MEF cells were treated with the pyrimidine synthesis inhibitor leflunomide (25 μM) 24 h prior to immunoblotting for protein expression. (**B**) WT MEF cells were treated with UV 15 J/m^2^ and incubated for 24 h (+/−) 0.2 mM nucleoside supplementation prior to immunoblotting for protein expression. (**C**) WT MEF cells were treated with 10 μM 5-FU and incubated for 24 h (+/−) 0.2  mM nucleoside supplementation prior to immunoblotting for protein expression. (**D**) WT MEF cells were treated with UV 20 J/m^2^ and incubated for 12 h prior to methanol extraction of nucleotides. Extracts were analyzed by LC-MS for relative nucleotide levels between UV-treated and untreated control cells, and untreated cell nucleotide levels were normalized to 100% for each experiment (Error bars represent the SEM n = 4). (**E**) *p53*^−/−^ MEF cells were treated with UV 20 J/m^2^ and incubated for 12 h prior to methanol extraction of nucleotides. Extracts were analyzed by LC-MS for relative nucleotide levels between UV-treated and untreated control cells, and the untreated cell nucleotide levels were normalized to 100% for each experiment (Error bars represent the SEM n = 4).

Increased nucleotide demand is one of the stresses present after extensive DNA damage, such as UV irradiation, and p53 can be activated under conditions of nucleotide shortage^31^. Our results show that PFKFB3 suppression occurs in response to 5-FU treatment (Fig. 1C,D), which inhibits nucleotide production^32^. Therefore, we hypothesized that p53-mediated *PFKFB3* suppression could be important when the cell requires more nucleotides. To test this hypothesis, we determined whether the pyrimidine synthesis inhibitor leflunomide could induce p53-dependent *PFKFB3* repression. Consistent with our hypothesis, treatment with leflunomide resulted in p53 activation and PFKFB3 repression in WT but not *p53*^−/−^ MEF cells (Fig. 4A). Moreover, nucleoside supplementation prior to UV-induced DNA damage reversed the suppression of PFKFB3 in both WT MEF (Fig. 4B) and p53 WT U2OS (Figure S4) cells without affecting p53 activation, indicating that nucleotide demand is a significant component of UV-induced, p53-dependent suppression of *PFKFB3*. Likewise, nucleoside supplementation prior to 5-FU treatment also reversed PFKFB3 repression in WT MEF cells, further supporting the hypothesis that p53 responds to nucleotide levels to repress *PFKFB3* expression (Fig. 4C). Because p53 appears to respond to nucleotide demand in response to UV and 5-FU treatment, the effects on ribonucleotide pools post-UV treatment were probed using LC-MS/MS to determine individual ribonucleotide levels relative to untreated control cells. Surprisingly, no clear effect was observed 6 h post-UV treatment in either WT or *p53*^−/−^ MEF cells (data not shown); however, WT MEF cells exhibited clear increases in ribonucleotide abundance 12 h post-UV treatment compared with untreated control cells (Fig. 4D). Importantly, this increase in ribonucleotide abundance was not observed in *p53*^−/−^ MEF cells, suggesting that p53, and presumably PFKFB3 regulation, play important roles in the maintenance of ribonucleotide pools in response to UV-induced DNA damage (Fig. 4E).

### Inhibition of *PFKFB3* expression augments PPP-dependent nucleotide production

**Figure 5:**
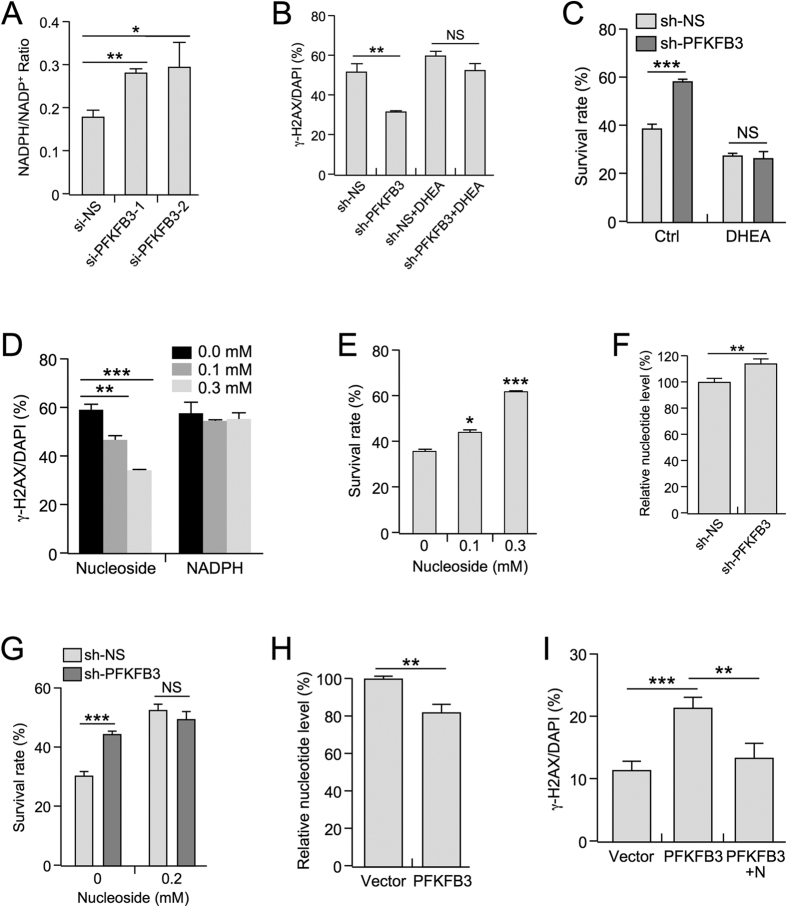
Inhibition of *PFKFB3* expression augments PPP-dependent nucleotide production. (**A**) NADPH/NADP^+^ ratios were determined in *p53*^−/−^ MEF cell lines stably expressing scrambled or sh-PFKFB3 constructs. (#1 p = 0.007 #2 p = 0.0341 n = 3 for each sample) (**B**) U2OS cells stably expressing scrambled or sh-PFKFB3 constructs were treated with QVD-OPh and (+/−) the PPP inhibitor DHEA (0.25 mM) for 30 minutes prior to 20 J/m^2^ UV treatment. After 48 h, the cells were fixed and stained for γ-H2AX and DAPI. (p = 0.0044 n = 3) (**C**) U2OS cells stably expressing scrambled or sh-PFKFB3 lentiviral constructs were treated with 40 J/m^2^ UV (+/−) DHEA (0.25 mM) for 24  h, after which the cells were trypsinized and counted using a Bio-Rad TC20 automated cell counter. (p = 0.0002 n = 3) (**D**) U2OS cells were treated with QVD-OPh and an equimolar nucleoside mixture or NADPH (0 mM, 0.1 mM, or 0.3 mM) for 30 minutes. Cells were then treated with 40 J/m^2 ^UV 48 h prior to fixation and then were stained for γ-H2AX and DAPI. (0.1 mM p = 0.0016;0.3 mM p < 0.001 n = 3) (**E**) U2OS cells were treated with a 0 mM, 0.1 mM, or 0.3 mM equimolar nucleoside mixture for 30 minutes, after which the cells were treated with UV 40 J/m^2^. Surviving cells were imaged and counted 24 h after UV treatment. (0.1 mM p = 0.0185;0.3 mM p < 0.0001 n = 5) (**F**) Nucleotide abundance was assessed by HPLC in *p53*^−/−^ MEF cells stably expressing scrambled or sh-PFKFB3 lentiviral constructs. (p = 0.01 n = 3) (**G**) U2OS cells stably expressing scrambled or sh-PFKFB3 constructs were treated (+/−) 0.2 mM equimolar nucleoside mixture for 30 minutes. After nucleoside treatment, cells were exposed to 40 J/m^2^ UV for 24 h, after which the surviving cells were counted by microscopy. (p = 0.0001 n = 5) (**H**) Nucleotide abundance was assessed by HPLC in *p53*^−/−^ MEF cells stably expressing GFP or PFKFB3-GFP. (p = 0.0242 n = 3) (**I**) HCT116 cells stably expressing GFP or PFKFB3-GFP were treated with QVD-OPh (+/−) 0.2 mM equimolar nucleoside mixture for 30 minutes. Cells were treated with 25 J/m^2^ UV 48 h prior to fixation and then were stained for γ-H2AX and DAPI (Vector vs. PFK p = 0.0002; PFK vs. PFK NT p = 0.0031 n = 5).

Reduced levels of PFKFB3 have previously been shown to decrease glycolytic flux, thereby resulting in the diversion of glucose through the pentose phosphate pathway (PPP)^33^. In agreement with this observation, we found that knockdown of *PFKFB3* in *p53*^−/−^ MEF cells increases the NADPH/NADP^+^ ratio, a known result of increased PPP production (Fig. 5A). To test whether the increase in DDR upon *PFKFB3* suppression is dependent on the PPP, we knocked down *PFKFB3* in U2OS cells and then treated these cells with dehydroepiandrosterone (DHEA), a potent inhibitor of the rate-limiting PPP enzyme G6PDH^34^. In support of the importance of augmenting PPP production in response to DNA damage, the protective effects of *PFKFB3* suppression on UV irradiation were abrogated by DHEA treatment with respect to DNA damage repair (Fig. 5B) and cell survival (Fig. 5C).

PPP products include NADPH and the nucleotide precursor ribose-5-phosphate. Thus, we determined whether exogenous supplementation of either component could rescue UV-induced DNA damage. Interestingly, only the supplementation of nucleosides exhibited a dose-dependent reduction of γ-H2AX foci observed in UV-irradiated U2OS cells, as supplementation with NADPH exhibited no effect (Fig. 5D). Consistently, nucleoside supplementation produced a dose-dependent increase in cell survival upon treatment with UV irradiation (Fig. 5E). Moreover, knockdown of *Pfkfb3* increased nucleotide levels by approximately 15% in *p53*^−/−^ MEF cells (Fig. 5F). Although PFKFB3 knockdown increased cell survival in response to UV irradiation, exogenous nucleoside supplementation abrogated this survival advantage, further supporting the idea that the enhanced survival observed after *PFKFB3* knockdown is due to increased nucleotide production (Fig. 5G). Conversely, exogenous overexpression of PFKFB3 resulted in an approximately 20% decrease in the relative abundance of all nucleotides (Fig. 5H and Figure S5), which is in agreement with reduced survival rates (Fig. 3G). Moreover, nucleoside supplementation was sufficient to abrogate the effect of PFKFB3 overexpression on DNA damage-induced γ-H2AX foci (Fig. 5I). Collectively, these results show that DNA damage-induced, p53-mediated *PFKFB3* suppression facilitates nucleotide production. Our results further suggest that p53-mediated *PFKFB3* suppression likely plays an important role in the diversion of glucose through the PPP to maintain *de novo* nucleotide production and facilitate DNA repair in response to UV irradiation.

## Discussion

In this study, we mined an inducible p53 microarray dataset and identified the novel p53 suppression target gene *Pfkfb3*, which plays a critical role in the coordination of glycolytic metabolism with the PPP and nucleotide production. Our study establishes a link between p53-mediated glycolysis suppression through PFKFB3 and increased *de novo* nucleotide production to generate the nucleotides necessary for DNA repair. Moreover, our results suggest that despite the expression of other DNA repair target genes, a lack of glycolytic regulation severely impairs DNA damage repair due at least in part to the lack of sufficient nucleotides.

**Figure 6:**
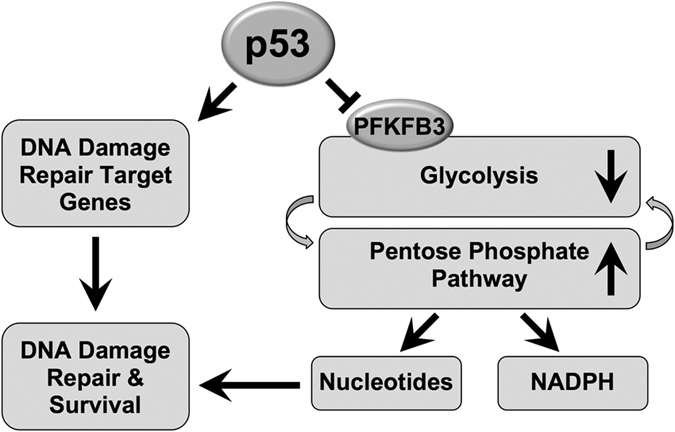
Model showing that p53 suppresses PFKFB3 expression, which results in increased *de novo* nucleotide production via the PPP to facilitate DNA damage repair and survival. Model representation of the role of p53 in the concomitant regulation of glycolysis, the PPP, and nucleotide production through PFKFB3 as well as through the regulation of DNA damage repair target genes to promote DNA damage repair.

In addition to the ability of PFKFB3 to increase F2,6BP levels, another p53-regulated target gene, *TP53-induced glycolysis and apoptosis regulator (TIGAR*), exhibits phosphatase activity toward F2,6BP. p53-dependent induction of TIGAR results in decreased F2,6BP levels, reduced glycolytic flux, and increased PPP activity, as TIGAR primarily counteracts PFKFB3 activity^14^. Our study suggests that by suppressing *PFKFB3* in response to UV irradiation and inducing TIGAR expression in response to other types of stress, p53 maximizes its control over glycolysis and PPP production. Although p53 regulates both PFKFB3 and TIGAR, it is noteworthy that high-level expression of PFKFB1, a homolog of PFKFB3 that also exhibits dominant kinase activity, has been shown to reverse TIGAR-induced PPP activity^14^. These results indicate that the kinase activity of PFKFB1 could override the phosphatase activity of TIGAR, which could explain the necessity for p53 to suppress *PFKFB3*. In response to acute stress, such as UV-induced DNA damage, the concomitant regulation of TIGAR and PFKFB3 could be necessary to rapidly and robustly produce the nucleotides required for repair. The dual regulation of an important rate-limiting step in glycolysis suggests that a major component of p53 tumor suppression involves the maintenance of nucleotide homeostasis through metabolic regulation (Fig. 6). Initially, the hypothesis that p53 is activated to upregulate PPP-dependent production of nucleotides seems to contradict previous research showing that p53 directly binds to and inhibits G6PDH, the rate-limiting enzyme of the PPP; however, it has also been noted that MG132- or doxorubicin-mediated p53 activation reduces the amount of p53-G6PDH binding^21^. This suggests that while inactivated cytoplasmic p53 may inhibit the PPP through protein-protein interaction, that p53 activation via genotoxic stress results in p53 trafficking to the nucleus to increase PPP activity through transcriptional regulation. Collectively, these data suggest a dynamic p53-dependent mechanism that depends on the sub-cellular localization of p53 protein.

UV irradiation generates cyclobutane-pyrimidine dimers and 6–4 photoproducts in the affected DNA, which requires nucleotide excision repair (NER) to maintain genomic integrity^35^. NER requires more nucleotides than base excision repair or non-homologous end joining, which repair DNA lesions without significant DNA synthesis. The high demand on nucleotide levels could explain why p53 regulates PFKFB3 specifically in response to UV irradiation. Whether p53 regulates PFKFB3 in response to other DNA damaging agents that generate DNA lesions that are repaired by other mechanisms will need to be addressed by future studies. Moreover, a sufficient and balanced nucleotide pool is required for proper cell division, as DNA replication in the presence of insufficient nucleotides leads to replication-induced DNA damage, which can result in mutations that lead to tumorigenesis^18^,^36^. Although a high degree of genomic fidelity is important in non-transformed cells, cancer cells require a degree of fidelity as well. Despite the adaptive advantages associated with increased genomic instability in cancer cells, too many mutations can be detrimental; therefore, even cancer cells must guard against mutation by maintaining *de novo* nucleotide synthesis through PPP upregulation. Consistent with the importance of the PPP in cancer, not only is PFKFB3 down-regulated in certain cancers but the rate-limiting PPP enzyme G6PDH is also commonly upregulated in cancers, suggesting that PPP flux is advantageous to cancer cell proliferation and survival^37^,^38^.

One specific area of therapeutic research that could benefit from our findings is the development of molecules that increase the efficacy of radiation treatment, known as radiosensitizers^39^. The efficacy of cancer radiation therapy is in part derived from the high proliferation rate of tumor cells, which results in more devastating replication-induced DNA damage in cancer cells compared with normal tissue. Based on the results of our study, we suspect that cancer cells in which the p53 stress response is disrupted may be especially vulnerable to PPP inhibition in conjunction with radiotherapy. Interestingly, multiple studies have shown that PPP inhibitors can sensitize cancer cells derived from difficult-to-treat tumors, such as gliomas and squamous carcinomas, to ionizing radiation^40^,^41^, suggesting that the development of additional PPP inhibitors could be a worthwhile endeavor. The potential value of PPP inhibitors as radiosensitizers is intriguing given the relative scarcity of nucleotides at any given moment in the cell and the importance of maintaining nucleotide levels sufficient for DNA repair. Our results provide the basis for future studies developing and investigating the efficacy of cancer therapeutics targeting the PPP.

## Materials and Methods

### Chromatin Immunoprecipitation Assay

U2OS cells expressing endogenous p53 were subjected to chromatin immunoprecipitation (ChIP) assays according the instructions recommended by the manufacturer (Quick ChIP kit, Novus Biological). Briefly, cells were treated with either 0 or 10 μM nutlin 12 h before crosslinking with 1% formalin. After cell lysis, the lysates were sonicated (Branson) to generate ~1000-bp fragments. Goat anti-human p53 FL-393 antibody and protein-A beads were used to immunoprecipitate p53-DNA complexes. Immunoprecipitated DNA was utilized as a template for PCR reactions consisting of 40 cycles of 95 °C for 30 seconds, 60 °C for 30  seconds, and 72 °C for 1 minute and analyzed with QuantStudio 6 Flex Real-Timer PCR System (Applied Biosystems) using the following primers:

P21 RE F 5′-CCACTGAGCCTTCCTCACAT-3′

P21 RE R 5′-TCTGACTCCCAGCACACACT-3′

PFKFB3 RE Intron F 5′-CCAGGCATGTTTCAGTTGAC-3′

PFKFB3 RE Intron R 5′-GTAATCCCATCTGCTGAGGTAGG-3′

### Measurement of Fructose-2,6-Bisphosphate Levels

Fructose-2,6-bisphosphate levels were determined based on the activation of pyrophosphate-dependent PFK1, as previously described^42^. Briefly, cells were pelleted by low-speed centrifugation and resuspended in a solution containing 20 volumes of 50 mM NaOH and 1 volume of 100 mM NaOH (pH 11.0) and vortexed for 10 seconds. The solution was then heated at 80 °C for 5 minutes before being placed on ice, and the solution was neutralized to pH 7.2 with cold acetic acid in 20 mM HEPES buffer. Sample extracts were then incubated at 25 °C for 2 minutes in a solution containing 50 mM Tris, 2 mM Mg^2+^, 1 mM Fru-6P, 15 μM NAD, 10 units/liter PP-dependent PFK1 enzyme, 0.45 kilounits/liter aldolase, 5 kilounits/liter triose phosphate isomerase, and 1.7 kilounits/liter glycerol-3-phosphate dehydrogenase (Sigma). Then, 0.5 mM pyrophosphate was added, and the rate of change in absorbance (OD 339 nm) per minute was measured for 5 minutes. The F-2,6BP concentration was calculated based on a calibration curve ranging from 0.1 to 1.0 pmol of purified F-2,6BP (Sigma) and then normalized to total protein content.

### Cell Culture and Reagents

U2OS cells were maintained in Dulbecco’s Modified Eagle Medium supplemented with 10% fetal bovine serum, 100 μg/ml penicillin, and 100 μg/ml streptomycin in the presence of 5% CO_2_ in a humidified incubator. Primary mouse embryonic fibroblast (MEF) cells were isolated on embryonic day 13.5 and were grown in a humidified 37 °C incubator in the presence of 5% CO_2_ and 3% O_2_ to simulate endogenous oxygen concentrations and to minimize oxidative stress. MEF cells were also maintained in DMEM supplemented with 10% FBS and 100 μg/ml penicillin-streptomycin. 4-hydroxytamoxifen (4-OHT) was purchased from Sigma. Mammalian protein extraction reagent was purchased from ThermoFisher Scientific.

### Plasmids and Adenovirus

The AdEasy XL system (Stratagene) was used to generate adenovirus constructs according to the instructions recommended by the manufacturer. Briefly, the full-length PFKFB3 cDNA (Open Biosystems) was amplified by PCR, cloned into pShuttle-CMV, recombined with pADEASY-1 vector, and transfected into 293 QBT cells to generate adenovirus particles. The pGL3 basic and pGL3 promoter vectors were utilized to subclone the identified p53RE from intron 1 of *PFKFB3* upstream of the firefly luciferase gene in each vector using the following insert oligos. The essential C and G bases in the putative p53RE highlighted in Figure S2 were mutated according to the oligo sequences shown below to generate the PFKFB3 p53RE mutant construct.

PFKFB3Luc-1F: 5′-CTAGCAGACAGAGTTTTGCTCTGTTTCCCAGGCTGGAGTGCATTGGTACAATCTCGGCTCACTGCAACCTCTGCCTC-3′ PFKFB3Luc-1R: 5′-TCGAGAGGCAGAGGTTGCAGTGAGCCGAGATTGTACCAATGCACTCCAGCCTGGGAAACAGAGCAAAACTCTGT CTG-3′ PFKFB3Lucm-1F: 5′-CTAGCAGACAAGTTTTGCTCTGTTTCCCAGGCTAGAGTGCATTGGTACAATCTAGGCTCACTGCAACCTTGCCTC-3′ PFKFB3Lucm-1R: 5′-TCGAGAGGCAAGGTTGCAGTGAGCCTAGATTGTACCAATGCACTCTAGCCTGGGAAACAGAGCAAAACTTGTCTG -3′

### Immunofluorescence Assays

Cells grown in a monolayer were fixed with formaldehyde, permeabilized with 0.2% Triton X-100, and stained with primary anti-phospho-H2AX (S139) antibody. After washing away unbound primary antibody, cells were incubated with AlexaFluor 594-conjugated goat anti-rabbit secondary antibody (Jackson ImmunoResearch Laboratories). 4′,6-diamidino-2-phenylindole (DAPI) was used for nuclear counterstaining. Immunostained cells were analyzed using an Olympus IX-81 microscope fitted with a SPOT camera and software.

### Luciferase Assay

Luciferase assays were conducted using the Dual Light Luciferase Assay kit from Applied Biosystems (Cat #BC100L) according to the protocol suggested by the manufacturer. Briefly, H1299 cells were plated in six-well plates 24 h prior to transfection with pGL3 basic (PFKFB3 mutant assay). The next day, cell extracts were prepared using 125 μL per well of lysis solution (100 mM potassium phosphate (pH 7.8), 0.2% Triton X-100, and 0.5 M DTT) after 2 PBS washes. Cell debris was removed by centrifugation, and 10 μL of the cell lysates were transferred to a 96-well plate in triplicate. Twenty-five microliters of Buffer A were added to each well and incubated at room temperature for 10 minutes before adding 100 μL of Buffer B. Luminescence was measured using a BioTek Synergy 2 plate reader. The plate was then incubated at room temperature for 1 h to decrease the luminescent signal before adding 100 μL of Accelerator II buffer and measuring the β-galactosidase signal using a BioTek Synergy 2 plate reader. Luminescence is shown as the relative firefly luciferase signal normalized to the β-galactosidase control.

### Antibodies

The following antibodies were purchased commercially: mouse anti-MDM2 2A10 (University of North Carolina Tissue Culture and Molecular Biology Support Facility), mouse anti-actin (Neomarkers), mouse anti-p53 DO.1 (Neomarkers), goat anti-p53 FL393 (Santa Cruz) and mouse anti-mp53 NCL-p53–*505* (Leica microsystems). Rabbit anti-PFKFB3 antibody (ProteinTech Group 13763–1-AP) and rabbit anti phospho-H2AX (S139) #9718S were purchased from Cell Signaling Technologies. Rabbit anti-p21 (C-19) was generously provided by Dr. Yue Xiong (UNC).

### DNA Damage Repair Efficiency Assay

Cells were treated with the caspase inhibitor QVD-OPh prior to additional treatments for 30 minutes. UV treatment (<40 J/m^2^ for U2OS cells and <15 J/m^2^ for MEF cells) was performed to induce DNA damage. Medium containing QVD-OPh and the indicated treatment was added, and the cells were incubated at 37 °C. Cells were then fixed 0, 24, and 48 hours after treatment and then stained for the DNA damage marker γ-H2AX^43^ and counterstained for DAPI. γ-H2AX-positive cells were counted and normalized to the total cell count as determined by DAPI staining.

### Lactate Assay

Extracellular lactate levels were measured using the Lactate Colorimetric/Fluorometric Assay kit according to the protocol suggested by the manufacturer (Biovision (Catalog # K607-100). Two microliters of culture medium were obtained from cells that were in culture for 24 hours. The absorbances (OD 570 nm) of the samples were measured using a BioTek Synergy 2 microplate reader.

### Measurement of Cellular Nucleotide Levels

An AKTA FPLC (GE Healthcare Life Sciences #18-1900-26) was used to quantify methanol-soluble extracts (Fig. 5). Five hundred-microliter samples were injected onto a Partisil 5 SAX anion-exchange column (4.6 mm × 250 mm; Whatman #4222-227). The nucleotides were separated using a gradient of 50% Buffer A (5 mM (NH_4_)H_2_PO_4_ pH 3.8) and 50% Buffer B (0.25 M (NH_4_)H_2_PO_4_, 0.5 M KCl pH 4.5) to 100% Buffer B for 30 min followed by an isocratic elution with 100% Buffer B for 15 min at a flow rate of 1.5 ml/min. The column was allowed to re-equilibrate to initial conditions for 5 min prior to the next injection. The absorbance of the nucleotides was determined at 280 nm.

### Measurement of Cellular Nucleotide Levels

Nucleotides were extracted from MEF cells using 100% methanol (Fig. 4). Briefly, cells were washed twice with PBS buffer before the addition of methanol, then the cells were scraped into an Eppendorf tube and vortexed. This extract was incubated on ice for 15 minutes prior to another short vortex, centrifuged at maximum speed for 5 minutes to remove cell debris, and dried using a speed-vac at room temperature. Dried extract was re-suspended in 60 μl of 1:1 acetonitrile/water solution containing 10 μg/ml of ^13^C-labeled standards (^13^C ATP, ^13^C CTP, ^13^C GTP, and ^13^C UTP (Sigma-Aldrich, St. Louis, MO, USA)), vortexed, and 10 μL was used for LC-MS analysis.

Liquid chromatography separation was conducted using an HILIC column (Venusil HILIC Column, 3 μm, 100 Å, 2.1 × 100 mm, Agela Technologies Inc., Wilmington DE, USA) optimized for both nucleotide, amino acid, and organic matter separation using 10-minute cycles based on the following solvent gradients. Solvent A: 100 mM ammonium acetate (Fisher Chemicals, Fair Lawn, NJ, USA), 20 mM ammonium hydroxide (Fisher Chemicals, Fair Lawn, NJ, USA) in HPLC grade water (Fluka, Sigma-Aldrich, St. Louis, MO, USA). Solvent B: 100% HPLC-grade acetonitrile (Fluka, Sigma-Aldrich, St. Louis, MO, USA). LC gradient: Starting with 98% Solvent B, 1 minute – starting gradient, 6 minutes – 50% Solvent B, 6.01 minutes – 2% Solvent B, 7 minutes – 2% Solvent B, 8 minutes – 98% Solvent B, 10 minutes – stop. Total flow rate of 0.4 ml/min (binary flow).

Mass spectrometry was performed in positive mode using an ABSciex 5600 with following parameters: Ion source gas 1–45, Ion source gas 2–30, Curtain gas – 20, Temperature – 450, Ion spray voltage – 4500 V, Declustering potential – 80 V, Collision energy 5 (35 – for product ion fragmentation). TOF mass detection 5 to 1050 Da. MS-MS was performed for the 10 most abundant products. Scheduled MRM was performed for 15 target metabolites (11 phosphonucleotides + 4 ^13^C-labeled internal standards). Relative nucleotide levels were calculated by measuring the peak area under the curve for each nucleotide species using ^13^C-labeled NTPs to account for ionization variance between runs. Valine was used to normalize for cell number, and 100% was defined based on the untreated control samples. Data were analyzed using Peakview software to determine nucleotide levels.

### Lentivirus-based shRNA and siRNA Treatment

Lentivirus-based shRNA constructs were purchased from Open Biosystems for human PFKFB3 (TRCN0000007338 NM_004566 RHS3979–9576297, TRCN0000007340 NM_004566 RHS3979-9576299, TRCN0000007341 NM_004566 RHS3979-9576300, TRCN0000007342 NM_004566 RHS3979-9576301) and mouse PFKFB3 (TRCN0000025414 NM_133232 RMM3981-9592822, TRCN0000025415 NM_133232 RMM3981-9592823, TRCN0000025416 NM_133232 RMM3981-9592824, TRCN0000025417 NM_133232 RMM3981-9592825, TRCN0000025418 NM_133232 RMM3981-9592826). shRNA constructs were cloned into the lentivirus-based pLKO.1 vector and were co-transfected into HEK293T cells along with the appropriate packaging vectors to produce infective virions. siRNA constructs targeting human and mouse PFKFB3 were as follows: siPFKFB3a: F-GCTGACTCGCTACCTCAACTT

R-TTCGACTGAGCGATGGAGTTG siPFKFB3b: F-TCTCCAGCCCGGATTACAATT

R-TTAGAGGTCGGGCCTAATGTT

### qRT-PCR

Total RNA was isolated from WT and *p53*^−/−^ MEF cells using the RNeasy Mini kit, and cDNA was generated using Superscript III reverse transcriptase. qRT-PCR was performed using SYBR green master mix in conjunction with a 7900HT Fast Real-Time PCR System according to the protocol suggested by the manufacturer. Relative expression was normalized to actin. The following primers were used: Fig. 1F mPFKFB3F: 5′-TGATGGTGGGGCTCCCAGCC-3′ mPFKFB3R: 5′-GTGGTCCTGCACTCTGTTCACC-3′ mActinF: 5′- GCCAGGACCATCAATGAAGTGGAG-3′ mActinR: 5′-GTTAGAGGTCGCTCTCGCCATAC-3′ mMdm2F: 5′-CCAACCATCGACTTCCAGCAGCATT-3′ mMdm2R: 5′-GATTGGCTGTCTGCACACTGGG-3′ mp21F: 5′-CCTGGTGATGTCCGACCTG-3′ mp21R: 5′-CCATGAGCGCATCGCAATC-3′ Fig. 1B,D mActin F 5′-CCACAGCTGAGAGGGAAATCGTGC- 3′ mActin R 5′-CCAGGATGGAGCCACCGATCC-3′ mPFKFB3 F 5′-CACTGCGTGAACAGGACAAG-3′ mPFKFB3 R 5′-TGGCGCTCTAATTCCATGAT- 3′

### Statistical Analysis

All statistical comparisons were performed using GraphPad 5.0 software based on two-tailed unpaired t-tests with n being greater than or equal to 3. The symbol * indicates a p-value < 0.05, ** indicates a p-value <0.01, and *** indicates a p-value < 0.001.

## Additional Information

**How to cite this article**: Franklin, D. A. *et al*. p53 coordinates DNA repair with nucleotide synthesis by suppressing PFKFB3 expression and promoting the pentose phosphate pathway. *Sci. Rep.* **6**, 38067; doi: 10.1038/srep38067 (2016).

**Publisher's note:** Springer Nature remains neutral with regard to jurisdictional claims in published maps and institutional affiliations.

## Supplementary Material

Supplementary Data
