# Supplementary material for: p53 coordinates DNA repair with nucleotide synthesis by suppressing PFKFB3 expression and promoting the pentose phosphate pathway
Source: Sci Rep. 2016 Nov 30;6:38067. doi: 10.1038/srep38067 (PMC5128917; doi:10.1038/srep38067)
Supplement: Supplementary Data [file srep38067-s1.doc]

**p53 coordinates DNA repair with nucleotide synthesis by suppressing PFKFB3 expression and promoting the pentose phosphate pathway**

Derek A. Franklin^1,2,*^, Yizhou He^1,3,*^, Patrick L. Leslie^1,3^, Andrey Tikunov^4^, Nick Fenger^4^, Jeff Macdonald^4^ and Yanping Zhang^1,2,3,5,¶^

^1^Department of Radiation Oncology and Lineberger Comprehensive Cancer Center

^2^Department of Pharmacology

^3^Curriculum in Genetics and Molecular Biology

^4^UNC Metabolomics Laboratory

School of Medicine University of North Carolina at Chapel Hill, Chapel Hill, NC 27514, USA

^5^Jiangsu Center for the Collaboration and Innovation of Cancer Biotherapy, Cancer Institute, Xuzhou Medical College, Xuzhou, Jiangsu 221002, China

- ^*^These authors contributed equally to this study

^¶^To whom correspondence should be addressed: ypzhang@med.unc.edu


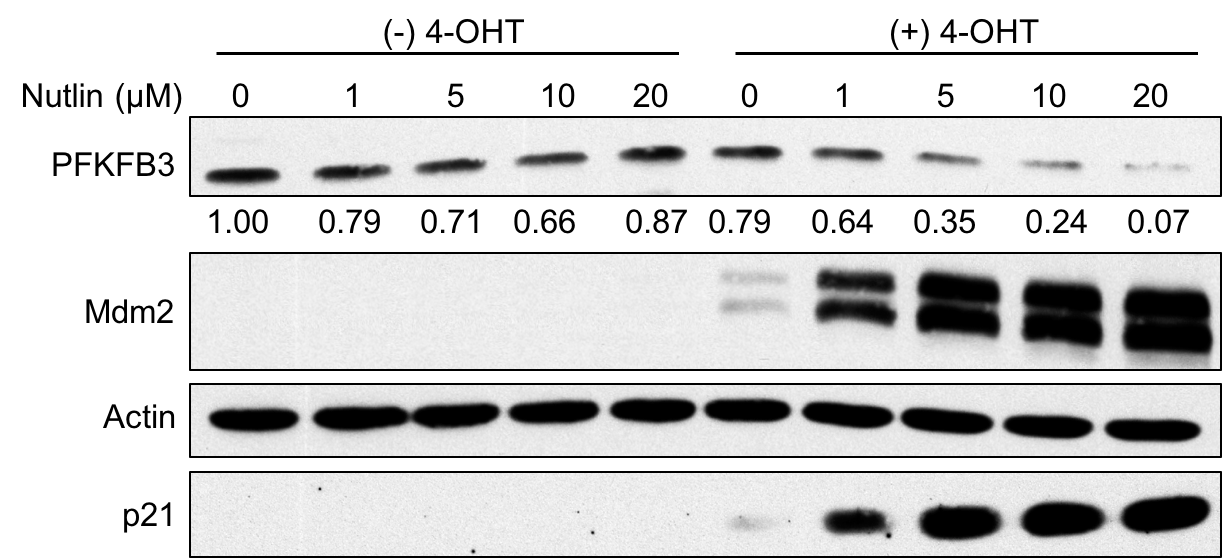


**Figure S1. PFKFB3 exhibits a dose-dependent decrease specifically in the presence of 4-OHT.**

*Mdm2^+/+^;p53^ER/-^* MEF cells were treated with or without 100 nM 4-OHT for 12 h followed by treatment with both 4-OHT and nutlin-3 at the indicated concentrations for 12 h. Cell lysates were analyzed by immunoblotting for indicated proteins.


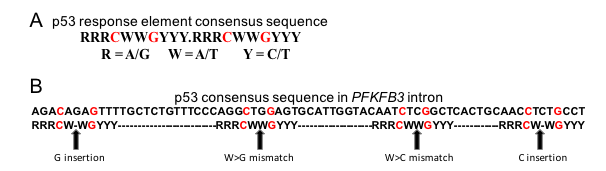


**Figure S2. Putative p53 response element in *PFKFB3* intron 1**

A. Conserved p53 response elements (p53RE) have been defined by the following sequence: RRRCWWGYYY (N_0-13_) RRRCWWGYYY (R=purine, Y=pyrimidine, W=adenine or thymine, N=any base) B. putative p53RE in the first intron of *PFKFB3* exhibits various alterations from this structure that may explain why p53 suppresses rather than activates this p53RE, which includes base pair insertions and A/T > C/G mismatches.


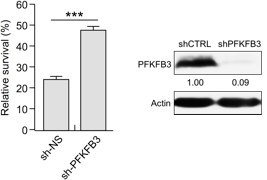


**Figure S3. Down-regulation of PFKFB3 increases cell survival.**

*Mdm2^+/+^;p53^ER/-^* MEF cells infected with lentiviral particles expressing scrambled (NS) or sh-PFKFB3 constructs were treated with UV 10 J/m^2^. Fresh medium was added after treatment, and the cells were incubated at 37°C for 24 hours prior to fixation and staining for DAPI to visualize the total number of nuclei present. A cell counting plugin for ImageJ was used to count surviving cells in each image. Western blot analysis of shCTRL and shPFKFB3 infected cells.


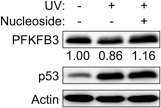


**Figure S4. Nucleoside supplementation reverses UV-induced suppression of PFKFB3 expression.**

U2OS cells were treated with UV 40 J/m^2^ and incubated for 24 h with or without 0.2 mM nucleoside supplementation prior to immunoblotting for protein expression.


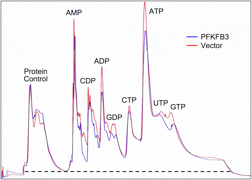


**Figure S5. Overexpression of PFKFB3 decreases nucleotide production**

*p53^-/-^* MEF cells infected with lentiviral GFP or GFP-PFKFB3 constructs were analyzed by HPLC for nucleotide abundance. When normalized for protein content detailed analysis shows that in PFKFB3 overexpressing cells nucleotide content is decreased with ATP and GTP exhibiting the largest decrease.
